# A Proposal for Value-Based Managed Entry Agreements in an Environment of Technological Change and Economic Challenge for Publicly Funded Healthcare Systems

**DOI:** 10.3389/fmedt.2022.888404

**Published:** 2022-06-16

**Authors:** Entela Xoxi, Filippo Rumi, Panos Kanavos, Hans-Peter Dauben, Iñaki Gutierrez-Ibarluzea, Olivier Wong, Guido Rasi, Americo Cicchetti

**Affiliations:** ^1^Postgraduate School of Health Economics and Management (ALTEMS), Università Cattolica del Sacro Cuore, Rome, Italy; ^2^London School of Economics and Political Science, London, United Kingdom; ^3^Rheinische Fachhochschule Köln, University for Applied Science, Köln, Germany; ^4^BIOEF, Public Foundation of the Department of Health to Promote Innovation and Research in Euskadi, Bilbao, Spain; ^5^Medi-Qualité Omega, Paris, France; ^6^Clinical Trial Center, Policlinico Universitario Agostino Gemelli IRCCS, Rome, Italy

**Keywords:** managed entry agreement (MEA), pharmaceutical pricing and reimbursement, value-based framework, innovativeness, registries, real-world data (RWD), value-based agreements

## Abstract

Managed entry agreements (MEA) represent one of the main topics of discussion between the European National Payers Authorities. Several initiatives on the subject have been organized over the past few years and the scientific literature is full of publications on the subject. There is currently little international sharing of information between payers, mainly as a result of the confidentiality issues. There are potential benefits from the mutual sharing of information, both about the existence of MEAs and on the outcomes and results. The importance of involving all the players in the decision-making process on market access for a medicinal product (MP) is that it may help to make new therapies available to patients in a shorter time. The aim of this project is to propose a new pathway of value-based MEA (VBMEA), based on the analysis of the current Italian pricing and reimbursement framework. This requires elaboration of a transparent appraisal and MEA details with at least a 24-month contract. The price of the MP is therefore valued based on the analysis of the VBMEA registries of the Italian Medicines Agency. Although the proposal focuses on the Italian context, a similar approach could also be adapted in other nations, considering the particularities of the single health technology assessment (HTA)/payer system.

## Introduction

The continuous challenge that drug regulatory authorities and HTA bodies in Europe are facing is that of guaranteeing patients quick access to new drugs, while ensuring the economic sustainability of the system at the same time. In a highly regulated and evolving market, there are different access and reimbursement models for new drugs. In recent years, flexible and diversified approaches have been developed to manage the entry into the market of new and high-cost health technologies, especially in rare diseases, capable of reconciling the financial and clinical aspects at the same time in a way that guarantees the sustainability of the system and the competitiveness of pharmaceutical companies.

In the context of regulatory clinical trials (CTs), variability in drug response is deliberately kept to a minimum by enforced treatment conditions and narrow selection criteria, which aim to restrict the patient population to high-responders and good-toleraters (1). As variability in drug response increases from CTs to outside label scenario, often average benefit–risk deteriorates as the result of the diminishing responsiveness to the beneficial effects and the increasing susceptibility to adverse drug effects, giving rise to the efficacy–effectiveness gap (a result of increasing variability of drug response owing to a combination of genetic, other biological and behaviors factors) (1). This explain the need for real-world data (RWD) to generate further evidence, to integrate those from CTs and confirm the drug benefit/ risk profile and consequently (at national level), to support the risk-sharing approach or managed entry agreement (MEA)/ conditional reimbursement.

MEAs are agreements between the pharmaceutical company and payers that allow conditional access to the market for innovative, high price medicinal products (MPs) with consequent modulation of the pricing and reimbursement schemes. Access and permanence of the MP on the market and the respective price and reimbursement (PR) conditions often depend, or are conditional, on the clinical evidence of the therapeutic benefits and/or actual costs once the MP is made available and/or on price agreements. Based on the typology of agreements registered in recent years (2, 3), the international literature reports two main categories: performance-based, risk-sharing agreements based on the outcome or expected clinical benefit of the new MP, or outcomes-based (OBMEA) and financial-based (FBMEA) agreements on budget impact uncertainty. Currently, MEAs represent one of the main topics of discussion between the European National Payers Authorities. Several European and global initiatives on the subject have been organized over the last few years (4–6). However, there is currently little international sharing of information between payers, linked mainly to confidentiality issues (7). Payers could benefit from mutual sharing of information, both on the existence of MEA and on the outcome of results (8–10).

### Objective

The aim of this study is to propose a new model of PR negotiation that overcomes the dichotomy between clinical trials and real-world data (RWD), here defined as a value-based MEA (VBMEA). The proposal is mainly aimed at the Italian context but is adaptable for some European HTA systems similar to the Italian one.

## Materials and Methods

In order to provide a contextual framework, non-systematic research was conducted to offer an overview of the following: the role of the Italian HTA authority; the criteria for the recognition of the innovativeness of a new MP; the role of registries on the MEAs in the Italian national setting; and the timing of the national PR procedures at present.

## Italian Medicines Agency

The Italian Medicines Agency (Agenzia Italiana del Farmaco, AIFA) is the national authority responsible for the regulatory, PR and health technology assessment (HTA) activities related to pharmaceuticals, including governance of pharmaceutical expenditure. It is supported by the Technical-Scientific Commission (Commissione Tecnico-Scientifico, CTS) and the Pricing & Reimbursement Committee (Comitato Prezzo e Rimborso, CPR). AIFA registries are an important operational part of the PR negotiation between AIFA and the pharmaceutical industry that constitutes a key element of the contract between the two parties. The MEAs implemented in the AIFA registries are also part of the PR contracts and therefore have the same validity as the registries (11). The peculiarity of these instruments is that they are not planned by the industry but are decided during the PR procedure by the Italian payer.

### Criteria on the Recognition of Innovation

The assessment of innovation status of an MP is an essential part of the PR decision in Italy (12). It is based on three criteria: unmet need, clinical added value and robustness of clinical evidence. As reported from Fortinguerra et al. (13) AIFA recognized the innovation taking into consideration the therapeutic indication-level: a five-point score (maximum to absent) plus a four-point GRADE score (high to very low). As established by the 2017 Budget Law, the recognition of innovation and its consequent benefits have a maximum duration of 36 months (also valid for the first-in-class). The permanence of the innovation status attributed to an MP will be reconsidered if there is evidence that justifies its re-evaluation. In any case, for conditionally/potentially innovative products, a re-evaluation at least 18 months from its grant is mandatory and the availability of new evidence that was positively assessed by the CTS may lead to “fully innovative” status, with the conferment of benefits for the remaining time. The “fully innovative” status is accompanied by inclusion in one of two funds (each of €500 million for cancer and other innovative MPs) and mandatory inclusion in the regional therapeutic formularies (as well as the “conditionally or potentially innovative” MPs) (12).

### AIFA Registries

**Figure 1:**
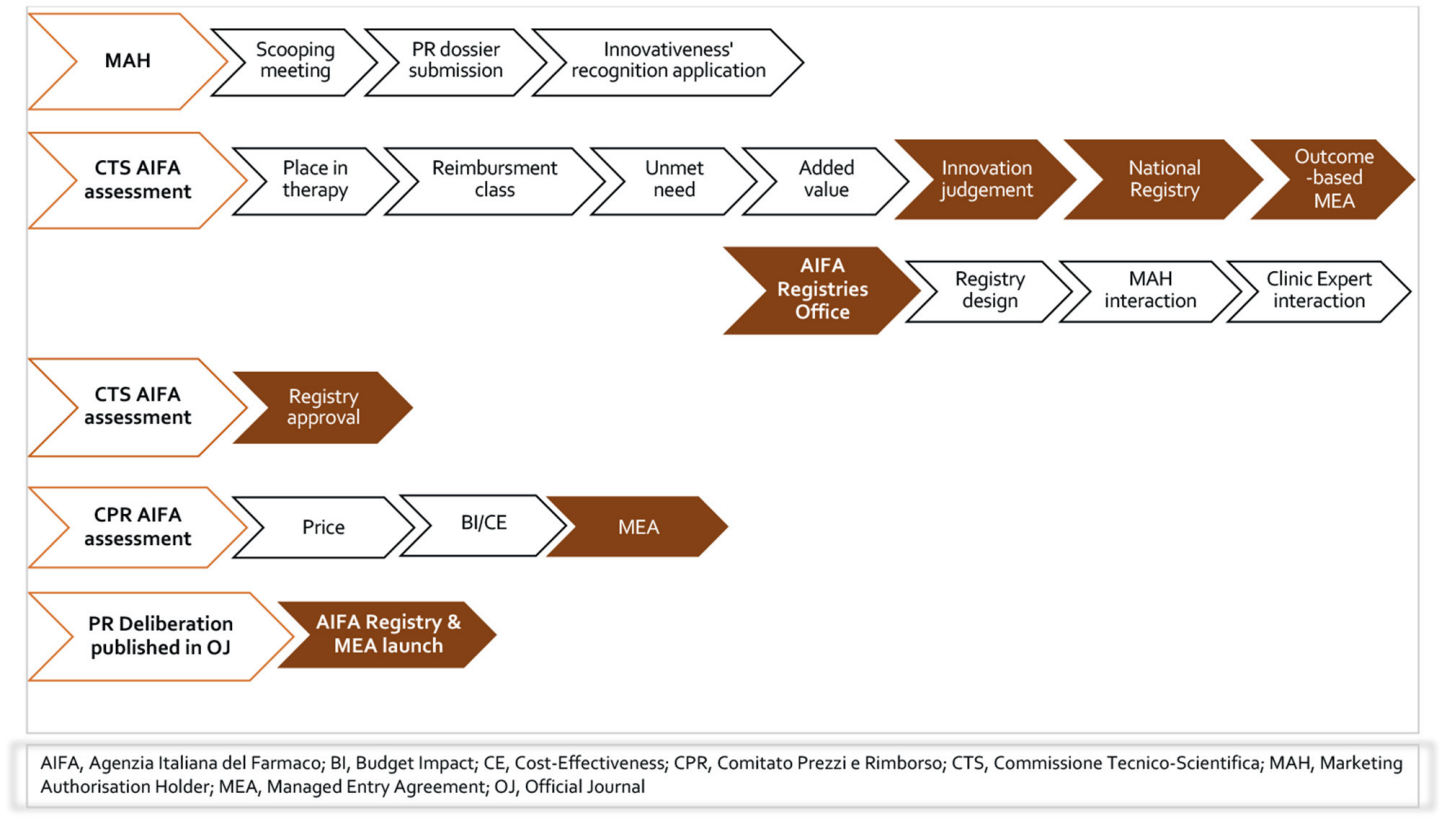
The flow of AIFA decision on individual-level registry with or without MEA.

Italy has a distinct system of national registries to support MEAs that has been in place for fifteen years, verifying the appropriateness of innovative hospital MPs with consideration of clinical as well as economic uncertainties to ensure best value for money (11). The Italian regulation recognizes registries as an integral part of the NHS information system, while the additional policies that were introduced for the purposes of attributing specific responsibilities for PR re-negotiation of medicinal products (14–17). For fully innovative products, AIFA registries are mandatory in order to manage pharmaceutical governance, and clinical and/or financial uncertainties. Figure 1 reported the flow of AIFA decision on individual-level registry with or without MEA.

Funded by pharmaceutical companies but fully governed by AIFA, the registries, despite the great debate on their administrative burden and lack of published results, continue to duplicate the current hospital data collection at the national level. Oncology remains the predominant therapeutic area, given the remarkable introduction of advanced therapies such as the CAR-T cell therapy for blood cancer (18, 19) and onasemnogene abeparvovec for SMA (20, 21) as well as agnostic-indications therapies as entrectinib (22) and larotrectinib (23) for the treatment of solid tumors with NTRK gene fusion. The AIFA registries have also been very valuable tools during the COVID-19 pandemic; in this regard, it is worth mentioning remdesivir for COVID-19 pneumonia (24) and monoclonal antibodies for the treatment of mild–severe COVID-19 disease (25–27).

Based on the last update of the National Report of Medicines Use in Italy, there are 2,655,909 patients whose treatment data are collected by 163 registries (28). In comparison to our last article related to the period until December 2019 (11), we note that 53 registries were closed in the period January 2020 to November 2021 as part of PR renegotiation processes (29).

### MEAs in Italy

Since 2005, AIFA has started to launch a wide range of MEAs for the management of clinical issues, budget impact uncertainty and inappropriate use of new, high-priced and potentially innovative MPs (11, 30, 31). The MEA management is done, in most cases and until 2017, through the use of national administrative appropriateness individual-level AIFA registries.

**Figure 2:**
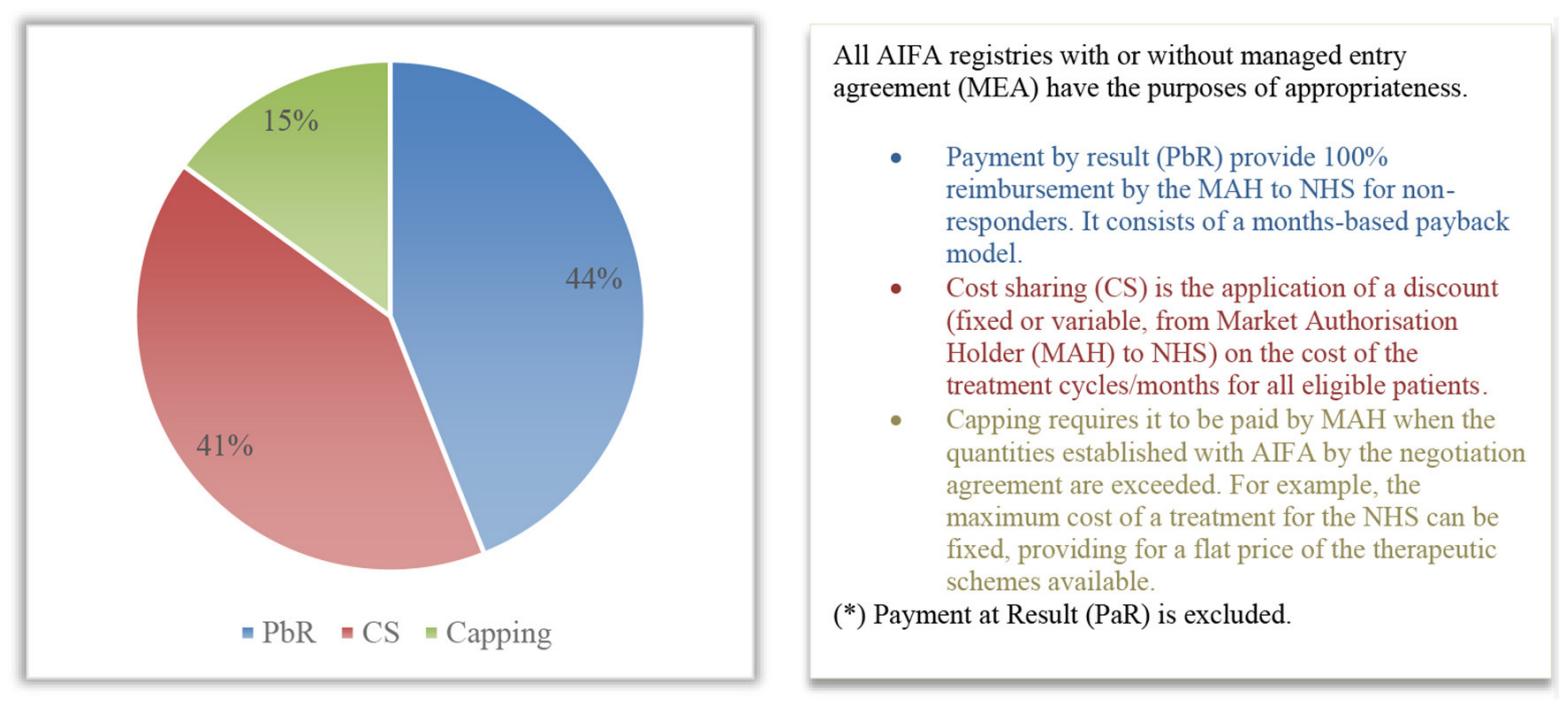
Distribution (%) of the types of MEA (*) patient-level for 2020 within AIFA registries.

The most widely applied agreement is Payment by Result (PbR), which, in 2020, constituted all the agreements based on the outcome (44% of the total agreements in force, corresponding to 20 product-based agreements) (Figure 2). In accordance with the paper by Xoxi et al. (11), there are no longer any active PbR agreements, neither risk-sharing nor success-fee. Cost-sharing financial agreements (19 agreements, equal to 41%) and 7 capping agreements (equal to 15%) follow in terms of frequency of MEA implementation within AIFA registries (Figure 2).

### Timing of the PR Procedures

In September 2021, AIFA published a preliminary result on the PR procedures concerning the timing of medicinal products authorization in 2018–2020 (32). Though the Italian times necessary for the evaluation, authorization for reimbursement and definition of the price of a medicine are to be considered satisfactory, when compared to European average times. In terms of time to availability, i.e., the time frame between the marketing authorization and the placing on the market for patients (corresponding, in most European countries, to the moment in which medicinals enter the reimbursement list), Italy is characterized by an average value of 418 days. Although this value is lower than the European average (504 days) it is very distant from countries such as Germany (120 days), Switzerland (166 days), Denmark (169 days), the Netherlands (213 days), Sweden (262 days), Austria (302 days), England (335 days), Russia (384 days) and North Macedonia (397 days). Furthermore, it should be specified that the AIFA report does not reflect on the critical issues of patient access to the regional and hospital level.

## Results

### The Rationale for a New Pathway

The procedure of access, reimbursement and price definition for new MPs, according to the current Italian regulation (33–35), focuses on examination of the therapeutic indication added value, the place in therapy and the therapy prices compared with the available alternatives, based on the clinical trial (CT) data.

**Figure 3:**
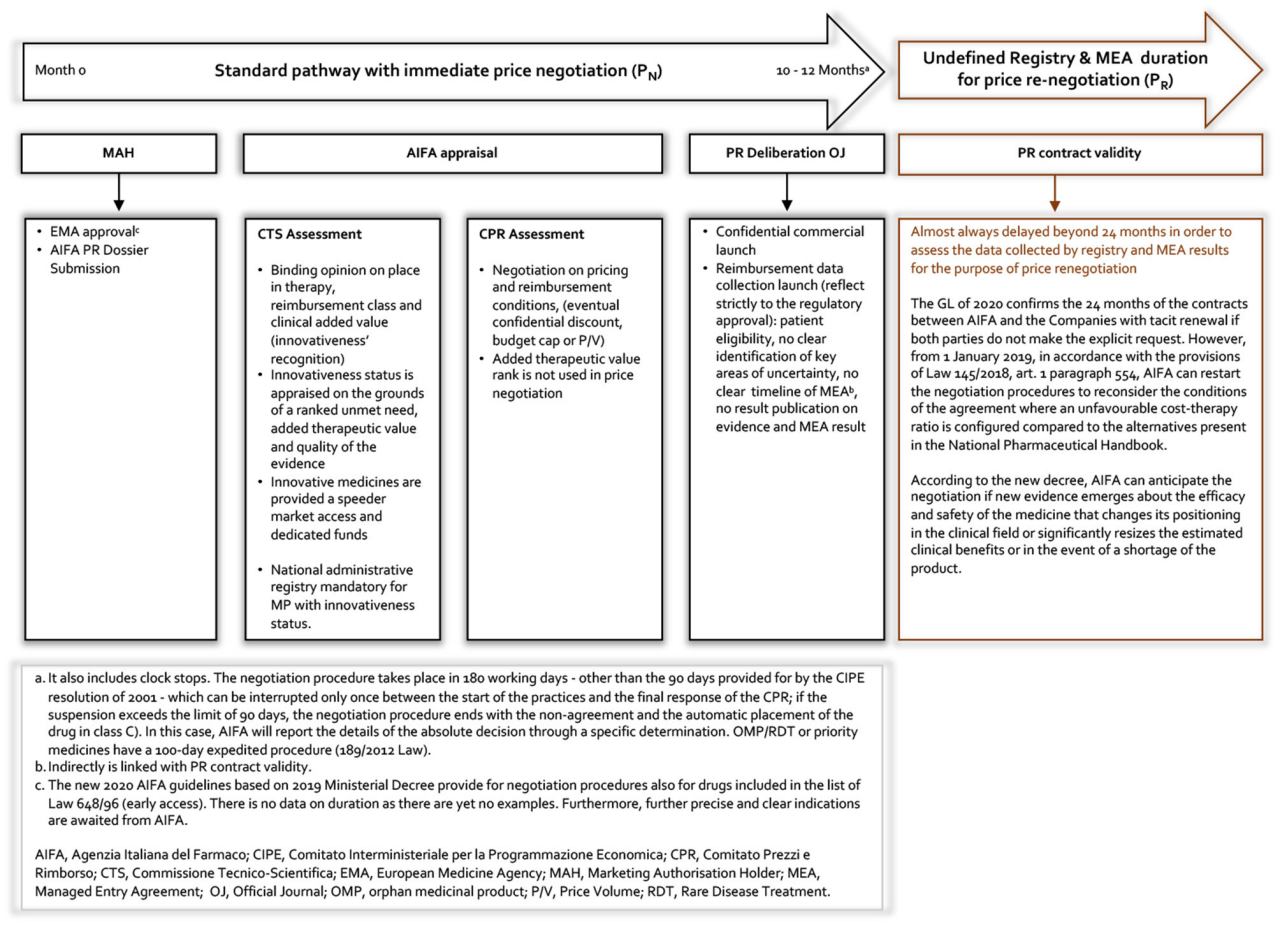
Current AIFA MEA registry pathway.

**Figure 4:**
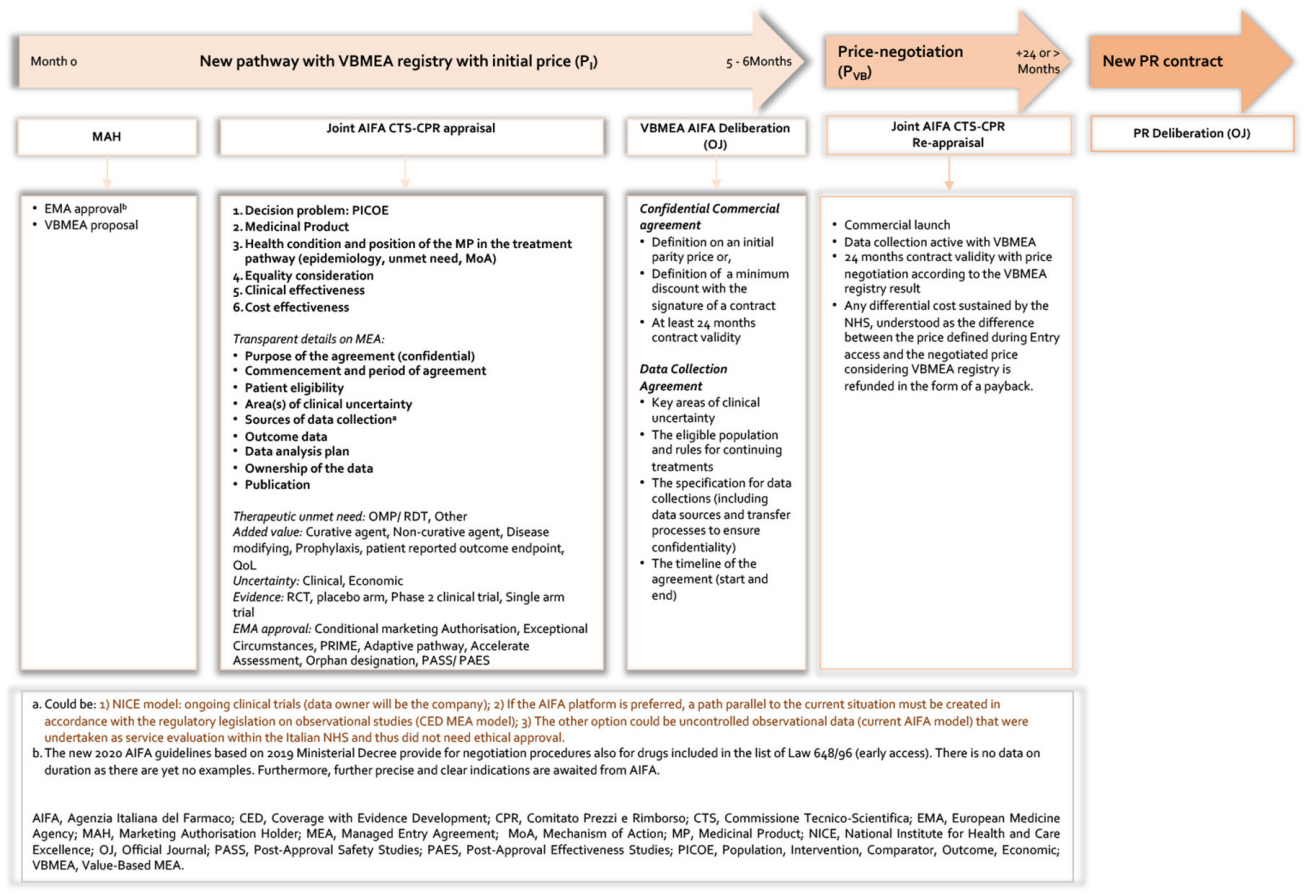
New pathway based on VBMEA AIFA registry.

Our proposal represents, here defined as a value-based MEA (VBMEA), a methodological approach joined with a new model of PR negotiation that overcomes the dichotomy between CT data and real-world (RW) data, by ensuring that the price and the negotiated conditions are controlled and verified through the AIFA registries platform. In Figures 3, 4, we summarize the characteristics of the current and new pathways. Currently^1^, the national debate is very animated on the new organizational model of AIFA, as well as on the operation of the two advisory committees. Regarding the European context, on 13 December 2021, the European Commission welcomed the adoption of the Regulation on Health Technology Assessment^2^ (to enter in force this year with applicability from January 2025) following its approval by the European Parliament. Additionally, the motivations for the proposed VBMEA are based on several facts. We explain our views and thoughts on this below. We believe that the key drivers of the proposal are value characterization, the time entry access, the registry data quality and the transparency approach.

### Value

Value-based pricing (VBP) implies that uncertainty of value should be considered when prices are set. Outcome-based MEAs (OBMEAs) could be implemented for this purpose, either based on population (i.e., using a post-marketing study to verify the medicine's impact in real life) or a payment-by-result contract, in which payers pay only for responders (36). Medicines are also issued approvals according to different indications. Indication-based pricing or OBMEAs should be implemented if the value differs across indications, as long as healthcare providers are able to track a medicine's use per indication. As Flume et al. (37, 38) stated:

“*First, one of the main concerns in VBP applications is how the benefit surplus generated by the drug might be unevenly distributed between the payers and the MAH, with great variation from case to case. As a result, companies may experience very high or very low returns on research and development costs, making their business case much more volatile. Second, VBP is defined on a single patient basis and is independent of volume, meaning that a VBP approach cannot take the size of the target population into consideration. Supporters of VBP would consider budget impact as a second-order driver, but budget constraints may actually be the most important determinant of price negotiation.”*

Italy negotiates drug prices only for binary decisions of approval or non-approval, not considering, during negotiations, whether there is a clear correlation between prices and benefits. Trotta et al. (39) evaluated whether there were better correlations between cancer drug prices and clinical outcomes in a setting where central price negotiations are mandatory for every new medicine. The study demonstrated that:

“*Correlation between drug costs and clinical outcomes was even lower than the ones previously noted in the US context, showing that negotiations did not tilt the relationship between drug prices and benefit positively. Thus, higher drug pricing remains despite the Italian legislative environment, where approval based on cost-effectiveness analysis and price negotiations have been mandatory by law since 2001.”*

#### Duration of Managed Entry Agreement Registries

By definition, if an AIFA registry is running, healthcare professionals (HCPs) must enter prescription data into the AIFA registry system in order to align with NHS reimbursement. Hence, the long duration of many of these registries has considerable administrative impacts in clinical practice.

Regarding the duration of the closed registries (32), we note a minimum of 0.8 years (lenalidomide label case for follicular lymphoma treatment) and a maximum of 15.2 years (bevacizumab for metastatic colorectal carcinoma). It is necessary to make a judgement on a case-by-case basis, but we can assume (especially for the registries launched up to 2017 and therefore before the AIFA Deliberation on the Recognition of Innovation) that the durations of registries constitutes an uncertainty that is not linked to the quality of the data or *ad-hoc* regulatory or economic evaluations. To date, we find no explanations of this.

The Law 6 August 2015, n. 125^3^ for the conversion of the Decree-law of 19 June 2015, n. 78, specifically reported:

“*In order to reduce the reimbursement price by the National Health Service of medicines subject to conditional reimbursement in the context of the AIFA monitoring registries, the benefits of which have elapsed, after two years from the market authorisation, are lower than those identified in the negotiation agreement, the Agency itself initiates a new negotiation procedure with the holder of the marketing authorisation pursuant to paragraph 33*.”

According to the law above and in response to criticism of the MEA registries' durations, the explicit delineation within an agreement between AIFA and pharmaceutical companies (as part of the contract between the two parties), that specifies, among the other details, the MEA starting and ending date (exit strategy) in the AIFA registries remains critical and very challenging.

#### The Impact of MEAs

In general, the return on investment of RWD registries with MEAs is multi-faceted and difficult to capture in full. The Italian case is especially complex, given AIFA's full management of the entire data platform and the application of the agreements. In its recent annual report (28), the agency published aggregated reports for MPs in some therapeutic areas of interest, such as chronic hepatitis C, age-related macular degeneration, family hypercholesterolemia (PCSK9), and non-small lung cell cancer ALK-inhibitors. They focused on the characteristics of the patients treated and their distribution in prescribing centers in the Italian regions. All these therapeutic areas relate to products that have a conditional reimbursement managed with the registry and MEA. But the efficiency of the MEA is unknown. In addition to this (and given that AIFA registry isn't an observational study) is the fact that the MEA captures only the data relating to the patients treated and registered in the AIFA registries, excluding the untreated patients.

Regarding the MEA reimbursements results (overall amount totalling €114,835,024), 73.3% of the MEAs obtained in 2020 relates to financial agreements, with 51.4% for cost-sharing and 21.9% for capping agreements. The payment for result agreements cover 26.7% of the MEA reimbursements. The MEA reimbursement percentages by ATC level (28) are instead distributed mainly across two categories: 83.5% for antineoplastic and immunomodulatory drugs (L), and 15.6% for general antimicrobials for systemic use (J). Then follow the MPs of the sense organs (S) with 0.6% of the MEA refund, the MPs of the nervous system (N) with 0.3%, and those of the musculoskeletal system (M) which represent 0.001% of the 2020 MEA refund.

Other conditional agreements implemented in Italy include those based on monitoring information flows of the expenditure and consumption (at the population level). These agreements, not based on the AIFA registry, are of a financial nature and can be mainly classified into expenditure ceilings by product and price/volume (P/V) agreements. In 2020, the MEA population level produced a total of €197.10 million. Specifically, €56.64 million was paid by pharmaceutical companies for the application of expenditure ceilings and the remaining €140.41 million for the application of P/V agreements. Considering the reimbursement class, €52.22 million was paid for class A products and €144.83 million for class H products (28).

In 2020, the total MEA reimbursements obtained by pharmaceutical companies, both managed through the registries (individual-level) and through other monitoring information flows (population-level), amounted to €343.7 million (28). The greater contribution is attributable to PV agreements (66.6%).

Some studies that highlight the effect of MEAs on the price of drugs (40) demonstrated that all agreements made during the establishment of the price for reimbursed MPs have allowed to lower the proposed price by 27.4% on average. The average P for these drugs was 32.2%. The average P for reimbursed orphan and non-orphan drugs were 25.1 and 28.6%, respectively. Recently, (7) evaluated the relation between oncology treatment costs and other information available at the time of the reimbursement decision in Italy and also explored the impact of confidential agreements on drug prices, analyzing oncology treatment costs using both list prices and confidential net prices reimbursed by the NHS (i.e., the price negotiated between AIFA and the pharmaceutical company). The results showed that, after price negotiation, a confidential discount was agreed upon for 91.4% of oncology drugs and further MEAs were established for 43.1% of the cases. In the univariate analysis, a significant relationship between the percentage variation of progression free survival (PFS) and the treatment cost was observed, regardless of the type of cost used (gross or net). This result was not affected by the existence of confidential agreements (gross cost, rho = 0.37 and *p* = 0.016; net cost, rho = 0.39 and *p* = 0.010) (7). The study showed that pricing negotiation tends to be associated with lower treatment costs when lower PFS gains were observed, particularly through greater use of MEAs in addition to simple confidential discounts. As has been mentioned in previous research, AIFA processes give the highest premium to treatments that demonstrate additional benefits in terms of OS. Otherwise, the results did not show a significant impact of the outcome of OS on reimbursed prices of recently approved oncology drugs (41).

However, AIFA must be recognized for their great effort in analyzing the data collected (including that of the MEAs) for decades, proceeding with the renegotiation of the price of products with MEA registries and proceeding with the closure of the registries, thus giving more options to their structure and especially to clinicians and pharmacists (32).

Our observation therefore raises three questions: what is the motivation for starting a MEA? What are the structural/fundamental elements of an MEA? What can be done with the results obtained and how can a possible exit strategy be planned? In Figure 3 we represent the current AIFA MEA framework. It should be clarified that, regarding the launch of the individual-level MEAs, this framework refers to the period up to 2017 as the AIFA (excluding the ATMPs) no longer initiates this type of agreement.

### Data Quality and Registry Design

Patient registries are organized systems that use observational methods to collect uniform data on a population defined by a particular disease, condition or exposure, and that is followed over time. The EMA has set up an initiative to make better use of existing registries and facilitate the establishment of high-quality new registries if none provide an adequate source of post-authorization data for regulatory decision-making (EMA). The initiative for patient registries, launched in September 2015, explores ways of expanding the use of patient registries by introducing and supporting a systematic and standardized approach to the benefit–risk evaluation of medicines within the European Economic Area (42). A number of challenges persist in using existing registries or establishing new ones, including: a lack of coordination between ongoing initiatives at national and international levels; harmonized protocols, scientific methods and data structures; data sharing and transparency; sustainability. These factors have led to inefficiency and a duplication of efforts. To address these problems, the EMA initiative seeks to create a European Union-wide framework on patient registries, facilitating collaboration between registry coordinators, such as physicians' associations, patients' associations and academic institutions, and the national agencies responsible for overseeing healthcare services, and potential users of registry data, such as medicines regulators and pharmaceutical companies (EMA). To support the initiative, the EMA has set up a cross-committee task force on registries, comprising representatives from EMA scientific committees and working parties, and experts from national authorities.

One of the main recommendations arising from the EMA workshop on patient registries (43) is that:

“*There is a need to provide rules to standardize data fields, data dictionaries and coding systems to improve data collection, quality and interoperability. In addition, where data from several datasets are combined, it is necessary to characterize the registry populations to understand endpoints, co-morbidities and safety concerns. It is recommended to decrease data collection paper forms and exploit current technology. User-friendly web-based platforms, use of mobile devices and user-friendly apps for providing feed-back information could increase participation of health care professionals, patients and families/parents. Technology may also facilitate the use of structured data (e.g., common endpoint definition and coding), data linkage, data pooling and data analyses.”*

Regarding data pooling and analysis, the document mentioned the European Society for Blood and Marrow Transplantation's (EBMT) experience in organizing operational and scientific support (i.e., programming and statistics) is an important component of good governance (43).

Another source of relevant experience is of the European Network of Cancer Registries (ENCR), supported by the European Commission, whose services developed an *ad-hoc* platform to facilitate harmonized data collection, as well as dissemination of aggregated indicators on cancer burden in Europe (incidence, mortality and survival) (43). The recent draft guideline (under public consultation) on registry-based studies addresses the methodological, regulatory and operational aspects involved in using registry-based studies to support regulatory decision-making, focusing on disease/condition registries to evaluate the benefit–risk profile of the MP (44). Among the various aspects considered in the guideline, the draft considers the acceptability argument of registry-based evidence for regulatory evaluation. On a case-by-case basis, objectives may include aspects such as the study of the natural history of disease, providing external or historical control data for clinical trials, evaluating the effectiveness and/or safety of an MP, and evaluating utilization of MPs.

From a HTA perspective, incorporating data from clinical practice into the drug development process is also a growing interest, since reimbursement decisions can benefit from methods which are able to estimate and predict the relative effectiveness of treatments at the time of product launch. A concrete example of where registries can provide clinical practice data is to support the building of predictive models that incorporate data from both randomized control trials (RCTs) and registries to bridge the efficacy–effectiveness gap; i.e., to generalize the results observed in RCTs to a RW setting. Collecting relevant HTA data in early development and planning post-authorization data collection, facilitated as needed by an early dialogue with industry, may therefore support rapid relative-effectiveness assessment and decision-making in drug PR. In this context, the EUnetHTA project has issued guidelines for the definition of the research questions and the choice of data sources and methodology that will support the generation of post-launch evidence by registries (45). The vision paper recommends that the Registry Evaluation and Quality Standards Tool (REQueST) requires infrastructure for its use that includes operational delivery; quality oversight, governance, methodological maintenance and development (including hosting the operational system); ownership and advocacy; and funding (45).

#### Registries as External Comparator for HTA

The importance of RWD lies in the transferability in real clinical practice of efficacy and safety data obtained from registration studies. There are various problems associated with this: variability between populations in clinical trials compared to real clinical practice settings; uncertainties regarding different outcomes in response rate, duration of the treatment, adverse events, etc.; risk of low therapy adherence; interference of other concomitant medicinal products taken for comorbidities; and potential different long-term tolerability profiles. Given the accelerated approval pathway, particularly for rare disease treatments, the number of MPs receiving marketing approvals based on data from non-randomized single-arm trials (SATs) has increased. According to Hatswell et al. (46), between January 1999 and May 2014, the EMA issued 795 approvals, including 44 solely on evidence from SATs, while the US Food and Drug Administration (FDA) issued 774 and 60, respectively in the same period. The interest is increasing in designs that use non-randomized control patients external to the CT for comparison reasons, to strengthen the evidence of a SAT or RCT (47, 48). The external comparator patients are not part of the same trial as those receiving the investigational product. They may be receiving the best standard of care treatment or be untreated, and may be sourced from previous trials, observational studies, registries or databases of routine healthcare. External comparators are increasingly used in regulatory decision making (47).

It is a natural next step that RWD should be proposed for use in establishing an external comparator arm in clinical trials (47, 49). Consequently, studies using RWD (now standard practice in post-authorization safety or effectiveness studies) can be more representative of the population requiring treatment in clinical practice (i.e., externally valid) (50). As reported by Gray et al. (51), there is a clear need for recommendations on how RWD external comparators are best used. Fears regarding a lack of predictability in regulatory requirements and rejection of non-standard methods remain real, despite publications from regulators such as the EMA concept paper on the extrapolation of safety and efficacy data across populations (51). Furthermore, the EMA advised in 2006 that historical RWD may be incorporated into the analytical framework through appropriate statistical methods (52). The Head of Medicines Agencies (HMA)/ EMA Joint Big Data Taskforce report provides recommendations regarding the regulatory acceptability of big data (53). In contrast, the FDA has agreeably endorsed the use of external comparator studies drawing on RWD in specific circumstances (54). We are aware that there are many challenges and gaps in understanding in this area, but, given the methods available (47), in our opinion, we believe that there is the potential to consider that AIFA registries could be used as external comparators for HTA purposes. Clearly, a detailed statistical analysis and strictly regulatory and legal examination is required, which is beyond the scope of this document.

### Transparency and Return on Scientific Evidence

There is a strong need to start taking advantage of the immense repository of AIFA data collected over the fifteen years that AIFA registries have existed. Theoretically, the registries generate evidence from RW clinical practice, representing an opportunity for collaboration among patients, academia, regulators, HTAs, payers and industry to undertake analyses that can support learning about health systems to improve patient outcomes. However, little has been done regarding this point (55) as they remain exclusive to the AIFA with little chance of interaction. It is not clear how the agency would or could collaborate with other stakeholders, especially with the academic domain. Recently, the AIFA, in collaboration with clinical or pharmacoeconomic experts, has published a variety of significant scientific papers on the effect of VBPs and MEAs on pricing negotiated by AIFA (7, 39, 40, 56–58). However, the procedures and criteria of collaboration with AIFA remain unknown. An attempt and a model of transparency on how to collaborate was made in 2015 in relation to the analysis of the natalizumab registry for the treatment of multiple sclerosis; unfortunately, it remained congested and did not see any progress regarding the intended plans (59).

Questions regarding this issue remain unanswered, such as: what is the return in terms of additional evidence generation? Why do some MEAs have such long durations and what knowledge is gained in such long period? What is the decision-support of these long MEAs? How has the use of registries and MEAs generated a positive impact on the sustainability of the healthcare system providing real support for value-based pricing? It is clear that the data collected and the MEAs are part of the standard PR renegotiations, but no results on the MEAs' efficiency and performance have been published so far, considering the confidentiality of the agreements present. These are the questions also posed in a recent article that analyzed the trend of the AIFA registries over the last 14 years (11). Furthermore, analysis of the AIFA registries could provide important scientific contributions in international peer-reviewed journals. The literature about challenges with MEAs in different jurisdictions is relatively large but there is a lack of published information about the factors that contribute to successful MEA systems and the constructs of MEAs for individual products. Confidentiality clauses are often responsible for the absence of published data in many countries, however there is important information about the constructs of MEAs that could be shared, including core datasets and aggregated analyses. Hence, AIFA has an important opportunity to continue its new programme of publishing reports (60–65) and open a transparent dialogue with stakeholders, which would also be useful for future strategy planning.

Lastly, and from a pharmacoepidemiological point of view, AIFA registries are not analytic studies, given their non-comparative nature, and so it is impossible to quantify the association between a drug's exposure and health phenomena, as they can't test the hypothesis of a causal relationship.

## Proposal for a New Pathway for VBMEAS

### Product Medicinal Launch With VBMEA Registry

The proposal is schematised in Figure 4 and, from a procedural point of view, it reveals three aspects: First, the possibility to organize joint CTS and CPR assessment in order to potentially partly reduce the time of negotiation process. The current legal basis for negotiating drug prices between AIFA and the companies dictates that the two AIFA advisory entities have specific and distinct roles. If the CTS (Technical-Scientific Commission) defines place in therapy, the level of innovation, reimbursement class and delimits the eligibility criteria for drugs subject to monitoring with the registries (including any performance-based risk-sharing agreement), that of the CPR (Pricing & Reimbursement Committee) is to negotiate the price medicines, also using any financial-based MEAs. We think that joint experiences as happened in the past (such as that of drugs for the treatment of chronic hepatitis C) can only speed up the decisive processes and inform the value of medicinal products in a more comprehensive and wide-ranging way. Secondly and most challenging (to date, there are no such information), the elaboration of a transparent appraisal from AIFA which is based on the decision problem (PICOE), the type of medicinal product that the company wants to be authorized and the health conditions under consideration. This also includes the position of the MP in the treatment pathways considering elements such as epidemiology, unmet need, and MoA. Moreover, it would be relevant to include in the appraisal equality considerations, clinical effectiveness and last but not least an assessment of the cost-effectiveness profile of the MP. Finally, the last is represented by a transparent MEAs process including registries characteristics (66, 67) which includes the following elements:

a. Purpose of the agreement (confidential) (MEA feasibility analysis including).
b. Commencement and period of agreement.
c. Patient eligibility (and why).
d. Area(s) of clinical uncertainty.
e. Sources of data collection (AIFA platform or other).
f. Outcome data (and timing).
g. Data analysis plan.
h. Ownership of the data.
i. Publication.

Thus, the VBMEA contract between AIFA and the relevant pharmaceutical company will include a confidential agreement with an initial price to be defined. The price should be defined according to the presence or absence of a comparator in the clinical practice and to direct or indirect comparison with the standard of care in the registration studies taking into account where an active substance or a placebo is used in the pivotal studies. The contract of VBMEA should be valid at least for 24 months: this range reflects the current AIFA guidelines (68). But clearly the area of uncertainty, type of drug (if rare disease) and above all the feasibility analysis of the implementation of the VBMEA must be taken into account.

Regarding the data collection agreement, AIFA and the relevant company should define the key areas of clinical uncertainty, the eligible population and rules for continuing treatments, and the specification for data collections. The latter includes data sources and transfer processes to ensure confidentiality. Finally, the time line of the agreement should be reported in the contract. Thus, the pharmaceutical company proposes to AIFA a 24-month contract with an ex-factory price (P_P_) equal to the cost in euros per syringe/vial/etc., and a transfer price to the National Public Health System (NPHS), following application of a confidential discount for public structures (-X%), of the cost in euros per syringe/vial/etc.

### Analysis of VBMEA Registry and Price Adaption

After 24 months, an analysis of VBMEA is carried out. The price of the MP is therefore valued based on AIFA's VBMEA registries. In this context, the novelty of the VBMEA would be represented by the fact that the cost value incurred by the NPHS, intended as the difference between the price in market (entry) access phase and the price negotiated (P_VB_) in light of the VBMEA results, shall be returned by the pharmaceutical company in the form of a reimbursement.

## Discussion

### Advantages for the System

The proposed VBMEA procedure has both advantages and disadvantages for both the AIFA and pharmaceutical companies.

The procedure will help to avoid increases in public expenditure due to the 24-month renegotiation based on the results from AIFA registries. Nowadays, there are three dynamic factors that influence the health system. The first two dynamics concern the aging of the population and the increase in people with chronic diseases. On the other side, there is constantly developing technological innovation. For this reason, to guarantee future innovation in a sustainable system, it is essential to find approaches and methods that do not result in increase of public spending.

Moreover, the introduction of a new pathway in MEA procedures to remodulate the initial negotiated price considering the RWD, combined with a reimbursement procedure for the differential costs incurred by the NHS, could help to inform decisions from the perspective of allocative efficiency. In fact, one of the main purposes of the VBMEA is precisely to better allocate the scarce resources available in the health system in such a way as to maximize the benefit in terms of health and quality of life for patients. Thus, in case of successful experimentation, the methodology may be adopted extensively to reimburse and renegotiate the price by using the 24-month AIFA registries results.

Finally, the procedure would ensure more transparency on price negotiation based on non-questionable RWD. Indeed, if it were possible to integrate an efficient monitoring system, guaranteeing the transparency of the acquired data, it would be much easier to justify policy decisions in the healthcare sector.

### Benefits and Advantages for Manufacturers

For pharmaceutical companies, the new procedure could avoid any delays to current market access and reduce the PR negotiation timing process. One of the crucial points for pharmaceutical companies and patients is timely access to innovation. Such an approach would speed up authorization procedures by making innovative and potentially more effective therapies available to patients. Furthermore, AIFA's refusal to adopt a new procedure does not involve any risk for the continuation of the procedure, according to the current methodology.

### Conditions for Successful Implementation

Both AIFA and pharmaceutical companies must commit to an important methodological effort to introduce a challenging new procedure. The pharmaceutical company has a need to make budget accruals for eventual payback, which this could represent a potential issue with regard to the budget for research and development of new drugs or therapies.

### Limitations of the Research

This proposal includes different limitations. The main limitation is represented by the possible reduction of the bargaining power of the CPR if the price negotiation is moved after the market launch of the MP. In fact, once the MP gets the reimbursement status, it will be much more difficult to negotiate lower prices or higher discounts, to the possible detriment of the NHS and the whole society.

Another limitation is related to the validity of RWD for at least 24 months. And here in particular consists one of the novelties of our proposal: that of looking carefully at observational studies rather than only administrative data which practically duplicate the enrolment criteria of patients in trials. In this context, it would be necessary to individuate the correct and feasible data collection process in such a way as to ensure transparent and continuous monitoring that can inform policy decisions (as mentioned in Figure 4, specifically Note a1–3). We can expect that the choice shown in Figure 4, Note a3 could be the most plausible one, given that the AIFA registries platform is already available and operational at the national level, even for the applications of more than a hundred agreements (FBA or PbR). We believe that a discussion should be opened with AIFA despite this to see the possibility of other data sources scenarios, such as a1 and a2 reported in Figure 4. This is also linked to the other critical issue of the system as a whole–that of duplicating data collection and the further administrative burden this causes.

Another issue could be represented by the individuation and involvement of the key expertise prescribing centers for the collection of data. It must be recognized that the current approach is well structured by AIFA and the regions. The concern in this case is the quality of the data and the entry of such data in the AIFA platform (Figure 4, Note a3) within the timeframe set in the agreement. It is not just a question of having NHS reimbursement (with a P_I_) but also of very specific timelines; this would therefore require a considerable effort by AIFA (but also by pharmaceutical companies) to clarify from the beginning with the centers (clinicians, pharmacists and health directors) the importance of the new pathway. Therefore, quality and exact timing are the key factors. With regard to the timing, this concern is also addressed to AIFA, to respect the timing for the analyses and start of the negotiation process (P_VB_) on the basis of the data obtained.

Another important point to consider is how the recognition of innovation can initially address data collection to which a VB agreement is closely linked. How can the AIFA's CTS judgement on the state of innovation according to the current approach be directed toward this new scheme; could it be case-by-case or in a systematic way? Which types of drugs (OMP, ATMP), which therapeutic areas (even if rare and/or severe and/or chronic disease), or which requirements already expressed in the three innovation criteria could be the most important drivers for this new approach? We have tried to define these variables in Figure 4, which also refers to international experiences or European projects that have extensively discussed the topic of MEAs. It is clear that the limitations in proposing a new, transparent VBMEA pathway (not only linked to the value of the single MP but also to the entire process of evaluation) is linked to the current legislation that regulates an AIFA MEA registry. We believe that the time has come to start an open discussion with the agency to review the legislative status, bearing in mind the current needs of the various stakeholders (including patients) and to start planning for a change in the near future.

Another limitation is the linkage between registries and digital healthcare. In the new era of healthcare digitalisation, the trend for value-based, patient-centric care provides an opportunity for person-generated digital health data to play a fundamental role in healthcare systems, and in the generation of RWE (69). If the regulators are a significant stakeholder in digital data and are being proactive in the areas of digital health through the publication of guidelines (70), more clarity also needs to be provided by payers and HTA bodies (71).

The current trend of AIFA MEAs must also be considered; to date, there are only payment at result for the ATMP (outcome-based on the success of the outcome) and only appropriateness registries for the other innovative MPs. It appears that AIFA has already partly found a strategy (not structurally explained) for products of greater complexity and very high prices by switching the PbR paradigm (based on therapeutic failure) to the new PaR payment model only in case of therapeutic success. It therefore remains to be seen how this will affect other medicinal products.

Finally, it is necessary to take into consideration the new European regulation on HTA and the long interim period necessary to set up the organizational framework adopting a series of implementing measures.

## Conclusions

Our proposal for a new VBMEA pursues the objective of providing a tool for improving the negotiation process, with the end goal of reducing approval times of drugs in the Italian healthcare context. In addition, if negotiation times were shorter, patients could access potentially innovative medicinal products sooner. We are confident that our proposal will serve to open a debate with the stakeholders involved, given the current European/national jurisdictive.

## Author Contributions

AC, EX, and FR contributed to conception and design of the study. EX wrote the first draft of the manuscript with the help of FR. AC organized an expert panels meeting involving PK, H-PD, IG-I, OW, and GR. The members of this panel contribute integrating the draft version of the manuscript. Following the feedback received, EX and FR drafted the final version of the paper. All authors contributed to the article and approved the submitted version.

## Funding

This research has been financed through an unrestricted educational grant of LEO Pharma S.p.A. LEO Pharma S.p.A. was not involved in the study design, collection, analysis, interpretation of data, the writing of this article or the decision to submit it for publication.

## Conflict of Interest

OW was employed by Medi-Qualité Omega. The remaining authors declare that the research was conducted in the absence of any commercial or financial relationships that could be construed as a potential conflict of interest.

## Publisher's Note

All claims expressed in this article are solely those of the authors and do not necessarily represent those of their affiliated organizations, or those of the publisher, the editors and the reviewers. Any product that may be evaluated in this article, or claim that may be made by its manufacturer, is not guaranteed or endorsed by the publisher.

## Footnotes

^1^ https://www.quotidianosanita.it/governo-e-parlamento/articolo.php?articolo_id=99749

^2^ https://ec.europa.eu/commission/presscorner/detail/en/ip_21_6771

^3^GU n.188 of 08/14/2015 – Ordinary Supplement n. 49.

## Abbreviations

AIFA: Italian Medicines Agency
Capp: Capping
CED: Coverage with Evidence Development
CIPE: Comitato Interministeriale per la Programmazione Economica
CS: Cost-Sharing
CTS: Commissione Tecnico-Scientifica
CPR: Comitato Prezzi e Rimborso
EMA: European Medicines Agency
FBA: Financial-Based Agreement
HTA: Health Technology Assessment
MAH: Marketing Authorization Holder
MEA: Managed Entry Agreement
MoA: Mechanism of Action
MP: Medicinal Product
NICE: National Institute for Health and Care Excellence
OBA: Outcome-Based Agreement
OD: Orphan Designation
OJ: Official Journal
OMP: Orphan Medicinal Product
PAES: Post-Approval Effectiveness Study
PaR: Payment at Result
PASS: Post-Approval Safety Study
PbR: Payment by Result
PBRSA: Performance-Based Risk-Sharing Agreement
PICOE, Population, Intervention, Comparator, Outcome: Economic
PR: Pricing and Reimbursement
P/V: price/volume
RDT: Rare Disease Treatment
RS: Risk-Sharing
VB: Value-Based
VBMEA: Value-Based Managed Entry Agreement
VBP: Value-Based Pricing.
